# Unveiling degenerative bone changes in the condyle: a texture analysis approach using cone-beam computed tomography

**DOI:** 10.1590/acb401325

**Published:** 2024-01-13

**Authors:** Michelle Bianchi-de Moraes, Natália Caroline Queiroz Costa, Gabriella Yasmim Santos da Silva, André Luiz Ferreira Costa, Fernanda Calvo Costa, Fernando Vagner Raldi, Sérgio Lúcio Pereira de Castro Lopes

**Affiliations:** 1 Universidade Estadual Paulista – Institute of Science and Technology – Faculty of Dentistry – São José dos Campos (SP) – Brazil.; 2 Universidade Cruzeiro do Sul – Postgraduate Program in Dentistry – São Paulo (SP), Brazil.

**Keywords:** Temporomandibular Joint Disorders, Jaw Diseases, Cone-Beam Computed Tomography

## Abstract

**Purpose:**

The degenerative joint disease is a temporomandibular disorder. By analysing texture parameters, it becomes possible to characterize and differentiate various tissues, based on their textural properties according to cone-beam computed tomography (CBCT). This study evaluated degenerative diseases in the temporomandibular joint through texture analysis.

**Methods:**

Eighty images of the jaw condyle with three types of degenerative diseases, flattening, osteophytes, erosion and control group were analysed, obtained through CBCT. The analyses were carried out through texture analysis with three regions of interest (ROIs) corresponding to specific bone sites. The scans were exported to MaZda software, in which the ROIs were delimited following previously marked contours, and the co-occurrence matrix values were calculated for selected texture analysis parameters.

**Results:**

The erosion group showed a significantly different behaviour from the other groups for all analysed parameters.

**Conclusion:**

This study highlights the potential of texture analysis in characterizing medullary bone changes in condyles affected by erosion. Texture analysis allows for a more comprehensive assessment of bone condition on CBCT images. These results have implications for early detection and monitoring of degenerative changes in the temporomandibular joint, thus allowing preventive intervention and personalized treatment planning, improving the prognosis of the disease.

## Introduction

The temporomandibular joint (TMJ) is one of the most complex in the body and consists of intracapsular and extracapsular components, such as capsules, ligaments, vessels, and nerves^1^^,^ 2. The intracapsular components can be divided into hard tissue components (i.e., condyle, mandibular fossa, and articular eminence) and soft tissue components (i.e., TMJ disc, and disc insertion)^2^.

The degenerative joint disease is a subtype of temporomandibular disorder (TMD) associated with joint disc displacement, trauma, functional overload, and autoimmune diseases^3^. TMJ bone changes may occur gradually after middle age, and hence it is also known as an age-related disease. The rate of TMJ bone changes is associated with the progression of degeneration: the first signs are erosion or flattening, whereas osteophytes and sclerosis are the last stage of degeneration^4^.

TMJ anatomy can be assessed by using imaging techniques, such as panoramic radiography, cone-beam computed tomography (CBCT), computed tomography (CT), and magnetic resonance imaging (MRI)^5^.

CBCT enables a more reliable assessment of bone components of the TMJ6. However, when characterized by small structural variations, the bone patterns affected by degenerative joint disease in the TMJ may not be identified in CBCT images.

Texture analysis (TA) is a method used to quantify spatial distribution of gray intensities in an image by using specific software^7^^,^ 8. In this context, TA provides an alternative in which quantitative features related to spatial distribution and patterns of pixels in CBCT images are extracted^9^^–^ 11. Some TA parameters can be used to differentiate fatty tissue in the bone marrow from mineralized tissues in the bone^12^. By analysing texture parameters such as entropy, contrast and homogeneity, it becomes possible to characterize and differentiate various tissues, including bone, dental structures, and soft tissues, based on their unique textural properties^13^.

The objective of this study was to investigate the possibility of initial detection of TMJ degenerative processes through CBCT images using TA. Specifically, the presence of flattening, osteophytes, and cortical erosions was investigated, which may not be easily detected at the beginning through visual inspection of CBCT images, in order to facilitate diagnosis prior to the worsening of degenerative processes.

## Methods

This retrospective study was approved by the human research ethics committee from the Institutional Review Board of Universidade Estadual Paulista “Júlio de Mesquita Filho”, according to protocol number CAAE 50377321.1.0000.0077, and was performed in line with the principles of the Declaration of Helsinki.

All stages of this study were carried out by two evaluators, previously calibrated. This calibration involved the following steps: the evaluators performed all described procedures, including the selection of sections, the delimitation of regions of interest (ROIs), and the TA, on this sample and repeated the steps at 15-day intervals, in imaging examinations covering 30% of the sample. The data obtained were subjected to intraclass correlation analysis, until a result corresponding to excellent intra- and inter-examiner agreement was obtained, being considered calibrated. The BioEstat 5.0 software (Sociedade Civil Mamirauá/Ministério da Ciência e Tecnologia, Conselho Nacional de Desenvolvimento Científico e Tecnológico, Belém, PA, Brazil) was used.

The sample size was calculated using the Raosoft software (http://www.raosoft.com/samplesize.html) in the study by Nussi et al.14, in which 20 individuals (TMJ) per group were identified, as necessary to obtain a 95% confidence interval and an alpha error of 5%.

Initially, 507 CBCT scans (1,014 TMJs) were obtained and submitted to two calibrated evaluators in CBCT.

**Figure 1:**
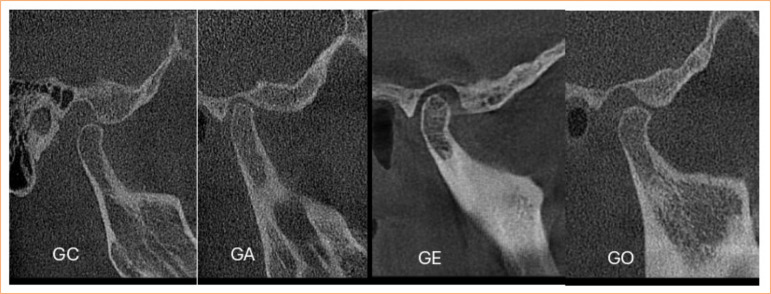
Computed tomography images showing condylar bone changes of group control (GC), group flattening (GA), group erosion (GE), and group osteophyte (GO).

Source: Elaborated by the authors.

Based on the inclusion and exclusion criteria, 80 TMJs were selected and then divided into four groups of 20 each, as follows (Fig. 1):

- Group C (control): without degenerative bone change in the condyle;
- Group A (flattening): condyle with a flat contour deviating from the normally convex shape;
- Group E (erosion): condyle with an area of discontinued or irregular cortical contour with decreased density;
- Group O (osteophyte): condyle with growths or projections on the margins.

The following inclusion criteria were applied:

- Images obtained with patients in maximum intercuspation;
- Images obtained with the occlusal plane parallel to the horizontal plane and the midsagittal plane perpendicular to it.

The exclusion criteria applied were:

- Images with artifacts and poor visualization of details;
- Low sharpness images;
- Images obtained by using another acquisition protocol;
- Images obtained from different scanners;
- Images obtained with identification of two or more joint degenerative changes.

### Image acquisition

All CBCT images were obtained on an i-CAT Next Generation cone-beam tomograph (Imaging Sciences International, Hatfield, PA, United States of America), with FOV protocol (Field of View of 6 × 16 cm covering the bilateral TMJ), with a voxel of 0.25 mm, in an average acquisition time of 14 seconds, 7 mA and 100 kV. All images were exported in their Digital Imaging and Communications in Medicine (DICOM) format to the OnDemand3D software (Cybermed Inc., Seoul, South Korea), in order to generate images for texture analysis. In the OnDemand3D software, the same window value (brightness and contrast) was applied to the images in the multiplanar reconstruction window in order to establish a single standard for all exams.

### Image analysis

With the curved tool, in the axial section in which the heads of the mandible were best visualized, its long axis was traced, going from its lateral pole to the medial pole. This cut then established latero-medial parasagittal cuts (perpendicular to the long axes drawn). Among them, three cuts were selected: the most central and two consecutive cuts, one more lateral, and the other one more medial, and they were exported in bitmap format (bmp) to the software MaZda 4.60 (Institute of Electronics, Technical University of Lodz, Poland), in which TA of the images were carried out.

In this software, the Draw Polygon tool was used to delimit the ROI with a diameter of 3 mm for all analyses, which was possible because the software allows the ROI to be saved as predetermined masks for later use with other images. This ROI was positioned in each slice for analysis of the central medullary, most anterior and most posterior regions of the condyle. The texture parameters of each of the three regions were extracted, and the average value was calculated and considered as an individual value for each patient.

### Texture analysis

TA is based on the co-occurrence matrix (MCO), which provides information about the spatial relationship between the pixels that make up the image and that are contained in the ROI, determined by the operator. The MCO is a tabulation of how many different combinations of pixel intensity values (gray levels) occur in an image. The MaZda 4.60 software can make variations in the coordinates of the spatial relationship of the component elements of this matrix, thus determining the frequency of different information relating to the values of the analysed pixels.

The MCOs were calculated for each pixel in the positions, S(1,0), S(0,1), S(1,1), S(1,-1), S(2,0), S(0,2), S(2,2), S(2,-2), S(3,0), S(0,3), S(3,3), S(3,-3), in four directions, horizontal (Horzl), vertical (Vertl), 45° (45dgr) and 135° (135dr).

The texture analysis was performed based on the MCO, and seven textural parameters were selected according to previous literature10,13, namely:

- Angular second moment (AngScMom): measurement of image uniformity;
- Contrast: represents the amount of local variation of gray level;
- Correlation (Correlat): linear measure dependence of gray level between neighboring pixels;
- Entropy: degree of disorder among pixels in the image;
- Difference of entropy (DifEntrp): difference of gray tones in image;
- Sum of entropy (SumEntrp): disorganization of the sum distribution of gray level;
- Sum of squares (SumOfSqs): measurement of the dispersion (related to average) of gray level distribution.

### Statistical analysis

Exploratory data analysis was performed by calculating summary measurements (mean, standard deviation, median, minimum and maximum). Comparison between groups was performed using the Kruskal-Wallis test with p-value correction using the false discovery rate (FDR) method. The significance level adopted was 5%. R software version 4.2.0 was used. Correction of the *p*-value by FDR was necessary because many comparisons (directions and parameters) were carried out, which increased the type I error of the work as a whole. When the p-value is corrected, the type I error is controlled.

## Results

In this study, 80 samples were evaluated, 20 in each group (flattening, control, erosion and osteophyte). The following tables show the results separated by parameter.

Through Table 1, it is possible to observe that, related to the parameter AngScMom, group E (erosion) presented higher values than groups A and O for position S(1,0) as well as higher values than group O for positions S(1,1) and S(2,-2).

**Table 1:** Results of multiple comparisons between groups regarding the parameter AngScMom*.

Position	*p*-value	Multiple comparison result
S(1,0)	**0.018**	Erosion > Flattening, Osteophyte
S(0,1)	0.050	
S(1,1)	**0.042**	Erosion > Osteophyte
S(1,-1)	0.051	
S(2,0)	0.151	
S(0,2)	0.151	
S(2,2)	0.086	
S(2,-2)	**0.026**	Erosion > Osteophyte
S(3,0)	0.151	
S(0,3)	0.143	
S(3,3)	0.151	
S(3,-3)	0.086	
Average	0.051	

* Significant *p*-values are indicated in bold.

Source: Elaborated by the authors.

In Table 2, it was observed that, regarding the parameter Contrast, group E presented lower values than the group O for positions S(1,0), S(1,-1), S(2,0), S(0,2), S(2,2), S(2,-2), S(3,0), S(0,3), S(3,3), S(3,-3) and average of all positions, as well as lower values than groups A, C (control) and O for position S(0,1). Furthermore, group E also presented lower values than groups C and O for position S(1,1).

**Table 2:** Results of multiple comparisons between groups regarding the Contrast parameter.

Position	*p*-value	Multiple comparison result
S(1,0)	**0.011**	Erosion < Osteophyte
S(0,1)	**0.001**	Erosion < Flattening, Control, Osteophyte
S(1,1)	**0.006**	Erosion < Control, Osteophyte
S(1,-1)	**0.010**	Erosion < Osteophyte
S(2,0)	**0.011**	Erosion < Osteophyte
S(0,2)	**0.006**	Erosion < Osteophyte
S(2,2)	**0.011**	Erosion < Osteophyte
S(2,-2)	**0.006**	Erosion < Osteophyte
S(3,0)	**0.011**	Erosion < Osteophyte
S(0,3)	**0.016**	Erosion < Osteophyte
S(3,3)	**0.006**	Erosion < Osteophyte
S(3,-3)	**0.011**	Erosion < Osteophyte
Average	**0.006**	Erosion < Osteophyte

*Significant *p*-values are indicated in bold. Source: Elaborated by the authors.

Table 3 describes that, in relation to the parameter Correlat, group E presented higher values than groups A and C for position S(0,1). Moreover, group E also presented higher values than groups C and O for position S(1,1).

**Table 3:** Results of multiple comparisons between groups in relation to the Correlat parameter*.

Position	*p*-value	Multiple comparison result
S(1,0)	0.100	
S(0,1)	**0.010**	Erosion > Flattening, Control
S(1,1)	**0.025**	Erosion > Control, Osteophyte
S(1,-1)	0.172	
S(2,0)	0.546	
S(0,2)	0.075	
S(2,2)	0.828	
S(2,-2)	0.592	
S(3,0)	0.592	
S(0,3)	0.230	
S(3,3)	0.247	
S(3,-3)	0.592	
Average	0.247	

* Significant *p*-values are indicated in bold.

Source: Elaborated by the authors.

In Table 4, it is observed that, in relation to the parameter SumofSqs, group E presented lower values than group O for positions S(1,0), S(0,1), S(1,1), S(1,-1), S(2,0), S(0,2), S(2,-2), S(3,0), S(0,3) and average of all positions. Moreover, group E showed lower values than groups C and O for positions (2,2) and S(3,3), as well as lower values than groups A and O for position S(3,-3).

In Table 5, it is observed that, in relation to the parameter SumEntrp, group E presented lower values than groups A, C and O for positions S(1,0), S(1,1), S(2,2). Group E also showed lower values than group O for positions S(0,1), S(2,0), S(2,-2), S(0,3), S(3,3), S(3,-3) and average of all positions, as well as lower values than groups C and O for position S(0,2).

**Table 4:** Results of multiple comparisons between groups in relation to the SumOfSqs parameter*.

Position	*p*-value	Multiple comparison result
S(1,0)	**0.007**	Erosion < Osteophyte
S(0,1)	**0.006**	Erosion < Osteophyte
S(1,1)	**0.004**	Erosion < Osteophyte
S(1,-1)	**0.007**	Erosion < Osteophyte
S(2,0)	**0.004**	Erosion < Osteophyte
S(0,2)	**0.015**	Erosion < Osteophyte
S(2,2)	**0.004**	Erosion < Control, Osteophyte
S(2,-2)	**0.011**	Erosion < Osteophyte
S(3,0)	**0.004**	Erosion < Osteophyte
S(0,3)	**0.004**	Erosion < Osteophyte
S(3,3)	**0.004**	Erosion < Control, Osteophyte
S(3,-3)	**0.004**	Erosion < Flattening, Osteophyte
Average	**0.004**	Erosion < Osteophyte

* Significant *p*-values are indicated in bold.

Source: Elaborated by the authors.

**Table 5:** Results of multiple comparisons between groups regarding the SumEntrp parameter*.

Position	*p*-value	Multiple comparison result
S(1,0)	**0.001**	Erosion < Flattening, Control and Osteophyte
S(0,1)	**0.009**	Erosion < Osteophyte
S(1,1)	**0.001**	Erosion < Flattening, Control and Osteophyte
S(1,-1)	0.169	
S(2,0)	**0.001**	Erosion < Osteophyte
S(0,2)	**0.011**	Erosion < Control and Osteophyte
S(2,2)	**0.032**	Erosion < Flattening, Control and Osteophyte
S(2,-2)	**0.001**	Erosion < Osteophyte
S(3,0)	0.077	
S(0,3)	**0.004**	Erosion < Osteophyte
S(3,3)	**0.025**	Erosion < Osteophyte
S(3,-3)	**0.014**	Erosion < Osteophyte
Average	**0.025**	Erosion < Osteophyte

* Significant *p*-values are indicated in bold.

Source: Elaborated by the authors.

Through Table 6, it was observed that, in relation to the parameter Entropy, group E presented lower values than groups A, C and O for positions S(1,0), S(0,1), S(1,1), S(1,-1) and S(2,0). Group E also showed lower values than groups C and O for positions S(0,2) and S(2,-2), lower values than group O for positions S(2,2) and (3,0), and lower values than group C for position S(0,3).

Table 7 shows that, in relation to the parameter DifEntrp, group E presented lower values than group O for positions S(1,0), S(1,1), S(1,-1), S(2,2), S(3,-3) and average of all positions. Moreover, group E presented lower values than groups A, C and O for position S(0,1), as well as lower values than groups A and O for positions S(0,2) and S(2,-2).

**Table 6:** Results of multiple comparisons between groups regarding the Entropy parameter*.

Position	*p*-value	Multiple comparison result
S(1,0)	**0.001**	Erosion < Flattening, Control and Osteophyte
S(0,1)	**0.001**	Erosion < Flattening, Control and Osteophyte
S(1,1)	**0.010**	Erosion < Flattening, Control and Osteophyte
S(1,-1)	0.001	Erosion < Flattening, Control and Osteophyte
S(2,0)	**0.010**	Erosion < Flattening, Control and Osteophyte
S(0,2)	**0.010**	Erosion < Control and Osteophyte
S(2,2)	**0.034**	Erosion < Osteophyte
S(2,-2)	**0.024**	Erosion < Control and Osteophyte
S(3,0)	0.023	Erosion < Osteophyte
S(0,3)	**0.023**	Erosion < Control
S(3,3)	**0.015**	Erosion < Flattening and Control
S(3,-3)	**0.028**	Erosion < Flattening and Osteophyte
Average	0.190	

* Significant *p*-values are indicated in bold.

Source: Elaborated by the authors.

**Table 7:** Results of multiple comparisons between groups in relation to the DifEntrp parameter.

Position	*p*-value	Multiple comparison result
S(1,0)	**0.013**	Erosion < Osteophyte
S(0,1)	**0.000**	Erosion < Flattening, Control and Osteophyte
S(1,1)	**0.001**	Erosion < Osteophyte
S(1,-1)	0.029	Erosion < Osteophyte
S(2,0)	0.214	
S(0,2)	**0.013**	Erosion < Flattening and Osteophyte
S(2,2)	**0.025**	Erosion < Osteophyte
S(2,-2)	**0.013**	Erosion < Flattening and Osteophyte
S(3,0)	0.062	
S(0,3)	0.400	
S(3,3)	0.074	
S(3,-3)	**0.013**	Erosion < Osteophyte
Average	0.013	Erosion < Osteophyte

*Significant *p*-values are indicated in bold. Source: Elaborated by the authors.

## Discussion

The present study investigated the role of TA applied to CBCT images of the TMJ for an objective and statistical evaluation of possible medullary condylar alterations caused by degenerative processes, such as presence of flattening, osteophytes, and cortical erosions. This technique was a new method of evaluating the presence of changes imperceptible to visual inspection of CBCT images and needed to be complemented by another modality of examinations, such as MRI. Seven texture parameters were used through MCO analysis following a study on CBCT images of TMJ using this technique for condylar evaluation with gender and age estimation^14^.

By analyzing the parameter AngScMom, our results indicated a statistically significant difference (*p* < 0.05) between group E (erosion) and group O (osteophyte group), with the former presenting higher values. It is known that the parameter AngScMom represents the number of gray levels in the image, being inversely proportional as the higher its value the smaller the number of gray levels, which reflects an image with greater uniformity^15^.

Analogously, it was observed that the parameter Contrast in group E showed statistically significant differences compared to groups A (flattening), C (control) and once again O (osteophyte), with lower values. In fact, a lower value of Contrast indicates a region with less noise and behavior of uniformity and similarity between gray levels.

A study carried out by Costa et al.^13^ evaluated the TA of CBCT images as a predictive method of bone quality for stability of dental implants, showing that regions with high contrast values indicated higher values of implant insertion torque and, consequently, greater implant stability. Thus, it is inferred that medullary bone with high contrast presents a structural trabecular network and medullary spaces with healthy characteristics, since they increase the chance of osseointegration, meaning that a high contrast value indicates non-homogeneous structures. In our study, the bone marrow of eroded condyles showed lower values than the other groups, which indicates homogeneity. This means that in this region of the bone marrow, in which there was loss of cortical content due to the erosive process, there is absence of trabecular bones, thus indicating a structurally simpler tissue, necrotic or edematous, with uniform composition.

Regarding the parameter Correlat, according to our results, there were statistically significant differences between group E compared to groups A (flattening), C (control) and O (osteophyte), with higher values. The parameter Correlat is the degree of interdependence between the pixels of the image, with higher values indicating greater interdependence between the pixels. Therefore, a greater uniformity of the tissue content represented by pixel values indicates no variation in the types of tissues analyzed in the region, with consequently less complex internal pattern^16^^,^ 17. Once again, it is inferred that the bone marrow of eroded condyles is poorly structured with unorganized content, such as a tissue resulting from an inflammatory (edema) or necrotic process.

In the study by Ito et al.^18^ using TA of CT images for evaluation of bone marrow and presence of mandibular osteonecrosis caused by bisphosphonate drugs indicated that higher values of the parameter Correlat were indeed associated with patients previously treated with these drugs and who actually had osteonecrosis, a finding corroborating our results. This difference occurred not only because the medullary bone of the mandible was evaluated instead of the mandibular condyles, as in our study, but also because TA was applied to fan-beam computed tomography images instead of CBCT ones.

The parameter SumOfSqs, according to our results, showed statistically significant differences between group E compared to groups A (flattening), C (control) and O (osteophyte), with lower values. Interestingly, the differences in group O involved three different positions, emphasizing that the behavior regarding the condylar medullary bone affected by erosion sharply differs from that of those with osteophytes. A lower value of the parameter SumOfSqs indicates less dispersion of grayscale image pixels and greater image uniformity^17^. Gonçalves et al.^10^ carried out a study using TA of CBCT images to differentiate bone regions affected by furcation lesions in upper molars from regions bordering these lesions and regions of healthy bone. Their results indicated that in regions of furcation lesions, in which there are an inflammatory process and bone disorganization with loss of normal architecture, the values of the parameter SumOfSqs were reduced in relation to healthy bone and bordering regions of the lesions. This indicates that this parameter is a significant marker for determining bone pathological processes when values are significantly lower than those of other groups.

According to the results of this study, it can be stated that the texture parameter SumOfSqs with lower values for group E indicates probable medullary pathological changes in this group. For Emshoff et al.^19^, condylar erosion is considered an inflammatory subset of processes (e.g., osteoarthritis), being a sign of progressive changes in these joint degenerative processes and potentially contributing to subchondral inflammatory processes, which leads to changes in dentofacial morphology or limited mandibular growth. The erosion process presents an advanced manifestation of joint degenerative processes causing exposure of the condylar medullary bone to the intracapsular environment, thus favoring the development of more acute inflammatory and necrotic processes, which justifies the more homogeneous behavior of the region evaluated by TA.

Considering the parameters related to entropy (i.e., SumEntrp, Entropy, and DifEntrp), which are complementary ones, group E once again presented statistically significant differences compared to groups A (flattening), C (control) and O (osteophyte) with lower values. These parameters indicate disorganization of the image pixels. According to the previously cited study by Costa et al.^13^, the parameter Entropy is directly linked to bone organization, with lower values indicating greater uniformity of the tissue with less variation in levels of gray (i.e., internal components).

Emphasis should be given to the finding that the behavior of group E was almost always differentiated from that of group O. Analysis showed that these two groups present antagonistic physiological behaviors, as in the case of osteophytic formation there is a progressive regenerative process of bone formation, which can somehow protect the underlying medullary bone. On the other hand, group E presents an enhanced consequence of a regressive alteration causing cortical destruction and medullary exposure. Our results point to the reflection of this behavior in the texture parameters.

This is a pioneering study as there is no other one in the literature addressing the methodology of evaluating the medullary bone of condyles for degenerative bone alterations by using TA of CBCT images, which makes our baseline task more difficult. However, the literature presents results related to the application of TA for evaluation of bone tissue in the stomatognathic system, on which we can base our work to interpret our results, which were coherently presented and compared to those already reported elsewhere^10^^,^ 13 ^,^ 14.

In the study by Queiroz et al.^20^, TA enabled to observe bone pattern changes in areas affected by osteonecrosis, thus increasing the precision in determining the real extent of osteonecrosis in the mandible related to medications. Also presenting positive results with the use of TA, Costa et al.^21^ concluded that TA allowed differentiating between odontogenic and non-odontogenic maxillary sinusitis on CBCT images by using the parameters of contrast, correlation and inverse moment of difference.

As other limitations of this study, the sample of each group could be increased to allow a greater base, including groups presenting more than one of the alterations evaluated here simultaneously, in order to compare the results of the influence of each alteration. Therefore, further studies could be conducted for this purpose.

## Conclusion

It was observed that the AT of the medullary bone of the TMJ condyles strongly indicated that group E presented a significantly different behavior in relation to the other groups in all parameters analyzed here, suggesting the presence of homogeneous tissue, which is indicative of necrotic tissue (avascular necrosis) or even inflammatory medullary edema in TMJs with cortical erosion. This fact becomes important, because it opens perspectives for evaluating the medullary bone in the TMJ using TA in CBCT images, which favors the diagnosis and prognosis of patients.

## Data Availability

All data sets were generated or analyzed in the current study.
