# ERRATA

**DOI:** 10.1590/acb401325.errata

**Published:** 2025-02-28

In the manuscript **Unveiling degenerative bone changes in the condyle: a texture analysis approach using cone-beam computed tomography**, which DOI is: https://doi.org/10.1590/acb401325, published in the journal **Acta Cirúrgica Brasileira**, 2025, v40, e401325:

Inclusion of an author:

André Luiz Ferreira Costa^2^

https://orcid.org/0000-0003-4856-5417

2. Universidade Cruzeiro do Sul – Postgraduate Program in Dentistry – São Paulo (SP), Brazil.

**Author’s Contribution:** Language Editing

